# The Complete Chloroplast Genome of *Baccaurea ramiflora* Lour. Cultivar Ha Chau (Phyllanthaceae, Malpighiales)

**DOI:** 10.1002/ece3.71145

**Published:** 2025-03-18

**Authors:** Pham Anh Thi Nguyen, Hanh An Nguyen, Hoang Dang Khoa Do, Do Tan Khang

**Affiliations:** ^1^ Department of Molecular Biology Institute of Food and Biotechnology, Can Tho University Can Tho City Vietnam; ^2^ Functional Genomics Research Center NTT Hi‐Tech Institute, Nguyen Tat Thanh University Ho Chi Minh City Vietnam

**Keywords:** Burmese grape, Dau Ha Chau, genomic evolution, *rps16* pseudogenization

## Abstract

*Baccaurea ramiflora* cultivar Ha Chau is a specialty fruit in Can Tho City, Viet Nam, and exhibits a potential economic value. However, its genomic data have not been reported. In this study, we sequenced and characterized the complete chloroplast genome of *B. ramiflora* cultivar Ha Chau using the Illumina sequencing platform, which has a quadripartite structure, including a large single copy (LSC), a small single copy (SSC), and two inverted repeat (IR) regions. The size of the genome was 161,378 bp (made up of 36.7% GC content), which was composed of an 89,801 bp LSC (34.3% GC), an 18,833 bp SSC (30.8% GC), and two 26,372 bp IR (42.7% GC) regions. The newly sequenced chloroplast genome contained 79 protein‐coding genes, 30 tRNA genes, and four rRNA genes. Notably, the *infA* gene contained an internal stop codon whereas only *rps16* exon2 remained in the complete chloroplast genome of *B. ramiflora* cultivar Ha Chau. Comparative genomic analyses indicated a high similarity of the chloroplast genome among *B. ramiflora* individuals regarding genome structure and gene content. However, *B. ramiflora* cultivar Ha Chau exhibited a lower pairwise identity in comparison with its counterparts. Further phylogenetic analysis also confirmed the sister relationship between *B. ramiflora* cultivar Ha Chau and other *B. ramiflora* individuals. This study provides initial genomic data for further studies examining evolutionary history, molecular markers, and genetic populations of *B. ramiflora* cultivar Ha Chau and its related species in Phyllanthaceae.

## Graphical abstract

This study reported the complete chloroplast genome of *Baccaurea ramiflora* cultivar Ha Chau. Comparative analyses revealed notable variations in the chloroplast genome of *B. ramiflora* cultivar Ha Chau, which contribute to tracing the evolutionary history of the Baccaurea genus and related species in the Phyllanthaceae family.

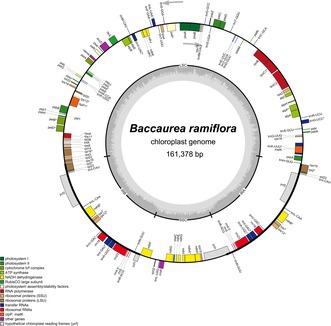

## 1 Introduction

*Baccaurea ramiflora* Lour., also called Burmese grape, is a member of the Phyllanthaceae family and is distributed from West India to East China (POWO 2024). Previous studies revealed different impacts of *B. ramiflora* (Nesa et al. 2018; Rohilla 2023; Roy et al. 2023). Specifically, the seeds of *B. ramiflora* were used to make biochar for wastewater treatment (Roy et al. 2023). Furthermore, the pulp and seed extracts of *B. ramiflora* exhibited anti‐inflammatory and anti‐diarrheal activities (Nesa et al. 2018). In fact, various chemical compounds such as alkaloids, saponins, and phenolic acids were found in different parts of *B. ramiflora* , which might contribute to its pharmaceutical properties (Rohilla 2023). Besides the implications, the genomic data of *B. ramiflora*, including nuclear and chloroplast genomes, were reported and used for exploring the evolution (Hu et al. 2021; Niu and Liu 2022; Huang et al. 2024).

In Viet Nam, *B. ramiflora* cultivar Ha Chau is strictly distributed in Phong Dien district, Can Tho City. Previous screening studies revealed similar bioactivities of *B. ramiflora* cultivar Ha Chau compared to other Burmese grapes (Xuan and Nguyen 2022; Xuan and Ha 2023). An additional genomic study based on *matK*, *rbcL*, *ycf1b*, *rpoC1*, *psbK‐I*, *atpF‐H*, and ITS regions indicated a high variation between *B. ramiflora* cultivar Ha Chau and other cultivars (Khang et al. 2022). Although a genomic study of *B. ramiflora* cultivar Ha Chau was conducted, there is still a lack of complete nuclear and organelle genomes that are essential for tracing evolution. Therefore, in this study, the complete chloroplast genome of *B. ramiflora* cultivar Ha Chau was sequenced and characterized using the Illumina sequencing method. Furthermore, comparative analyses among *B. ramiflora* individuals were conducted to elucidate the evolution of chloroplast genomes. The outcomes of this study provide initial genomic data for further studies examining nuclear and mitochondrial genomes of *B. ramiflora* cultivar Ha Chau as well as developing molecular markers for the identification of Burmese grape cultivars.

## 2 Materials and Methods

**FIGURE 1:**
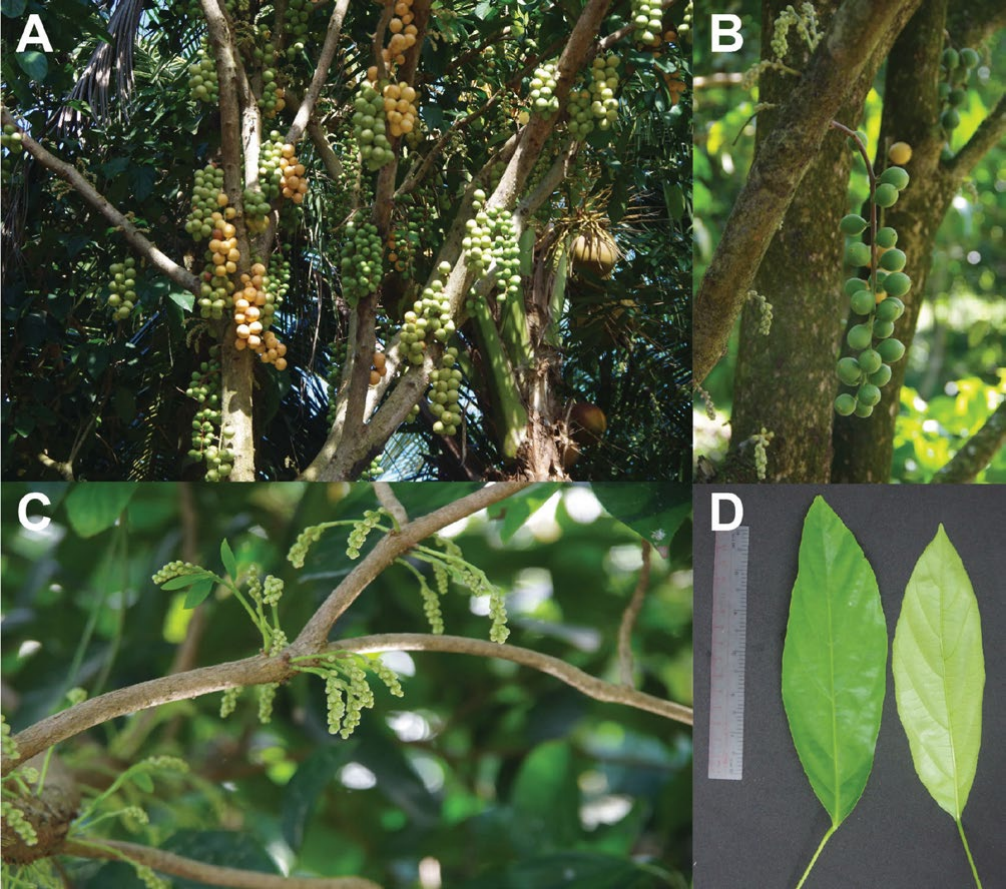
Illustration of *Baccaurea ramiflora* cultivar Ha Chau in the field. (A) The whole plant in fruiting season. (B) The fruits. (C) The inflorescences. (D) The leaves. The tree is up to 10 m tall. Leaves are oblong with green adaxial surface and yellowish‐green abaxially surface. Flowers are yellowish‐white and cluster on pistillate inflorescences emerging from old branches or trunk. Fruits are green or yellow at mature stage in ovoid or subglobose shape. Photo taken by Nguyen Pham Anh Thi in Phong Dien district, Can Tho City, Vietnam.

The healthy leaves of *B. ramiflora* cultivar Ha Chau were collected at Phong Dien District, Can Tho City, Vietnam (geographical location 9°57′47.5″N 105°37′26.4″E, Figure 1). Permission by the local authority was not needed to collect samples of *B. ramiflora* . Then, the collected leaves were stored in a deep freezer at −81°C for further experiments. The specimen of *B. ramiflora* cultivar Ha Chau was deposited to the Institute of Food and Biotechnology under voucher number CTU‐IFB‐202401001 (contact person: Dr. Nguyen Pham Anh Thi, email: npathi@ctu.edu.vn). The collected leaves were used to extract total genomic DNA using a modified CTAB method (Schenk et al. 2023). The quality of the DNA sample was checked using NanoDrop One Microvolume UV–Vis Spectrophotometer (Thermo Fisher Scientific, USA) for the minimum concentration of 100 ng/μL and 1% agarose gel electrophoresis with a clear band on the gel without smear. Then, the qualified DNA sample was applied for the Nextseq500 (Illumina, USA) to generate a dataset of 150 bp paired‐end reads.

The quality of raw reads was checked using fastp v0.23.4 with default settings to eliminate reads with a phred score ≤ 20, reads containing N bases, and reads shorter than 100 bp (Chen et al. 2018). The filtered reads were used to assemble the complete chloroplast genome using NOVOPlasty v4.3.5 with the reference genome of *B. ramiflora* (accession number PP497828) and default settings (Dierckxsens et al. 2016). The newly complete chloroplast genome of *B. ramiflora* cultivar Ha Chau was annotated using Geseq with default settings (Tillich et al. 2017). The protein‐coding regions were checked for start and stop codons using Geneious Prime v2024.1, whereas tRNAscan‐SE was used to verify tRNA composition (www.geneious.com; Chan and Lowe 2019). The map of the plastome was illustrated using OGDRAW with default settings (Greiner et al. 2019). The pairwise identity of the chloroplast genomes of different *B. ramiflora* individuals was calculated using Geneious Prime v2024.1 with the default settings.

For phylogenetic analysis, complete chloroplast genomes of 20 species were downloaded from the NCBI database (https://www.ncbi.nlm.nih.gov/), which include 18 species of Phyllanthaceae and two species of Euphorbiaceae as outgroups (Table 1). A total of 77 protein‐coding genes (except *infA* and *rps16* due to pseudogenization) of 21 species were extracted from the chloroplast genomes and aligned using MAFFT with default settings (Katoh 2002). The aligned sequences were then applied to jModeltest2 to find the best substitution model with default settings (Darriba et al. 2012). Consequently, the TVM + I + G was selected as the best substitution model for the dataset of 21 examined species. Phylogenetic analysis was conducted using maximum likelihood (ML) and Bayesian inference (BI) methods. For the ML method, IQ‐TREE2 was used with the TVM + I + G substitution model, 1000 replications for bootstrap analysis, and other default settings (Minh et al. 2020). For BI methods, MrBayes v3.2.7a was used with the TVM + I + G model, 1,000,000 generations, and a discard of 25% of samples (Ronquist et al. 2012). The Figtree v1.4.4 was used to illustrate the phylogenetic trees (http://tree.bio.ed.ac.uk/software/figtree/).

**TABLE 1:** List of species for phylogenetic analysis.

Family	Subfamily	Species	Accession number	*rps16* status
Phyllanthaceae	Antidesmatoideae	*Bischofia polycarpa*	MZ826267	Intact
		*Antidesma bunius*	ON022043	Intact
		*Aporosa dioica*	OR637442	Exon2 remained
		*Baccaurea ramiflora*	PP497828	Exon2 remained
		*Baccaurea ramiflora*	MT900598	Exon2 remained
		*Baccaurea ramiflora*	MW717296	Exon2 remained
		*Baccaurea ramiflora*	ON881834	Exon2 remained
		*Baccaurea ramiflora* cultivar Ha Chau	PQ469841	Exon2 remained
	Phyllanthoideae	*Flueggea virosa*	OP580983	Intact
		*Phyllanthus amarus*	NC_047474	Intact
		*Phyllanthus urinaria*	OL693862	Intact
		*Glochidion eriocarpum*	PP073972	Intact
		*Breynia androgyna*	PP059845	Intact
		*Emblica officinalis*	MN122078	Intact
		*Synostemon bacciformis*	OR637441	Intact
		*Cathetus clarkei*	OR683690	Intact
		*Leptopus cordifolius*	MZ424188	Exon1 remained
		*Sauropus spatulifolius*	NC_058216	Intact
		*Bridelia tomentosa*	MW357611	Lost
Euphorbiaceae		*Euphorbia hirta*	MW429224	Lost
		*Mallotus paniculatus*	MZ597547	Intact

## 3 Results and Discussion

**FIGURE 2:**
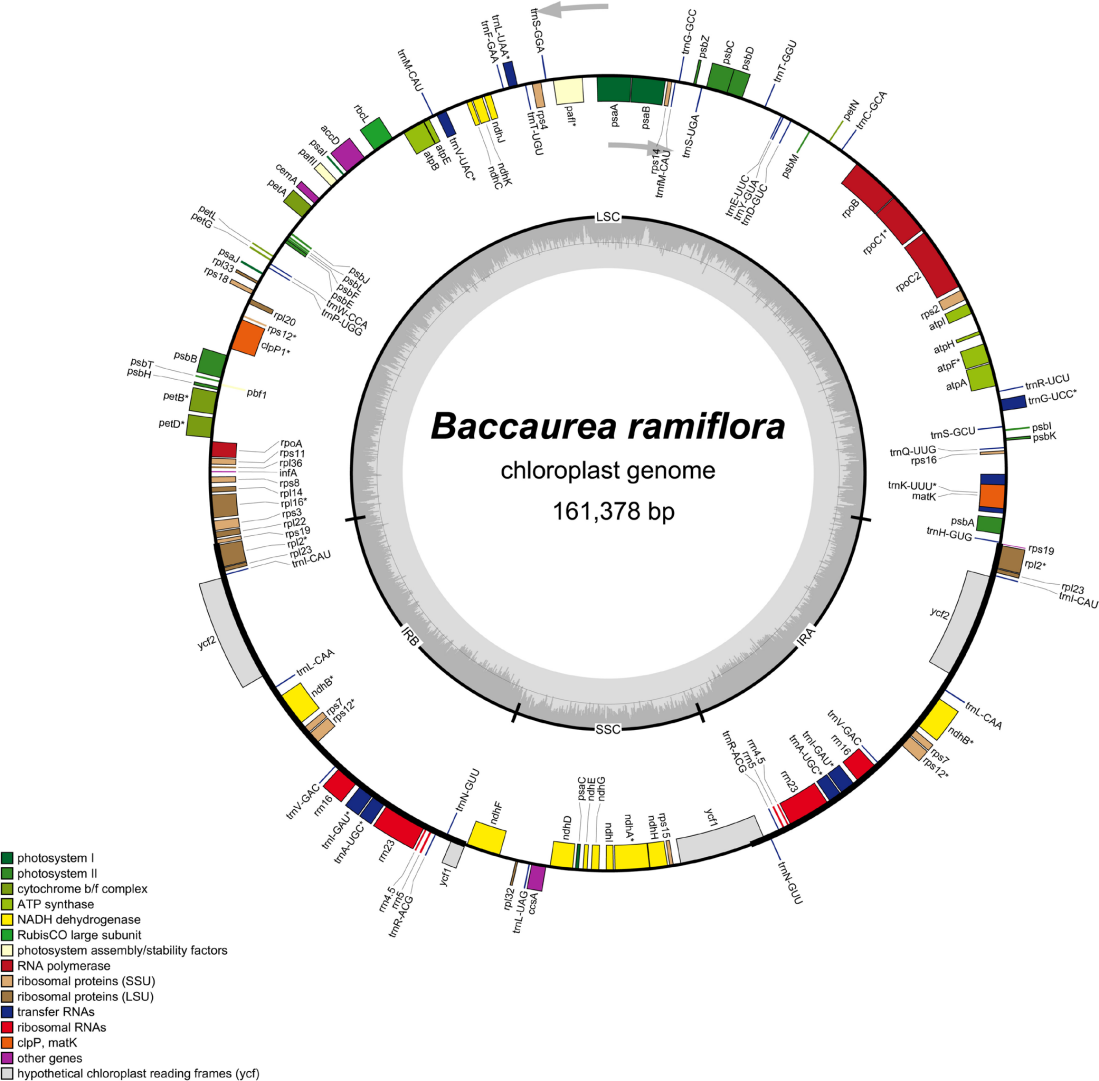
Map of the chloroplast genome of *Baccaurea ramiflora* cultivar Ha Chau. The arrows indicated the translation directions of inner and outer genes. The inner circle indicates the GC contents. The asterisks mean the gene having intron. IRA and IRB, Inverted repeat regions; LSC, Large single copy; SSC, Small single copy.

**FIGURE 3:**
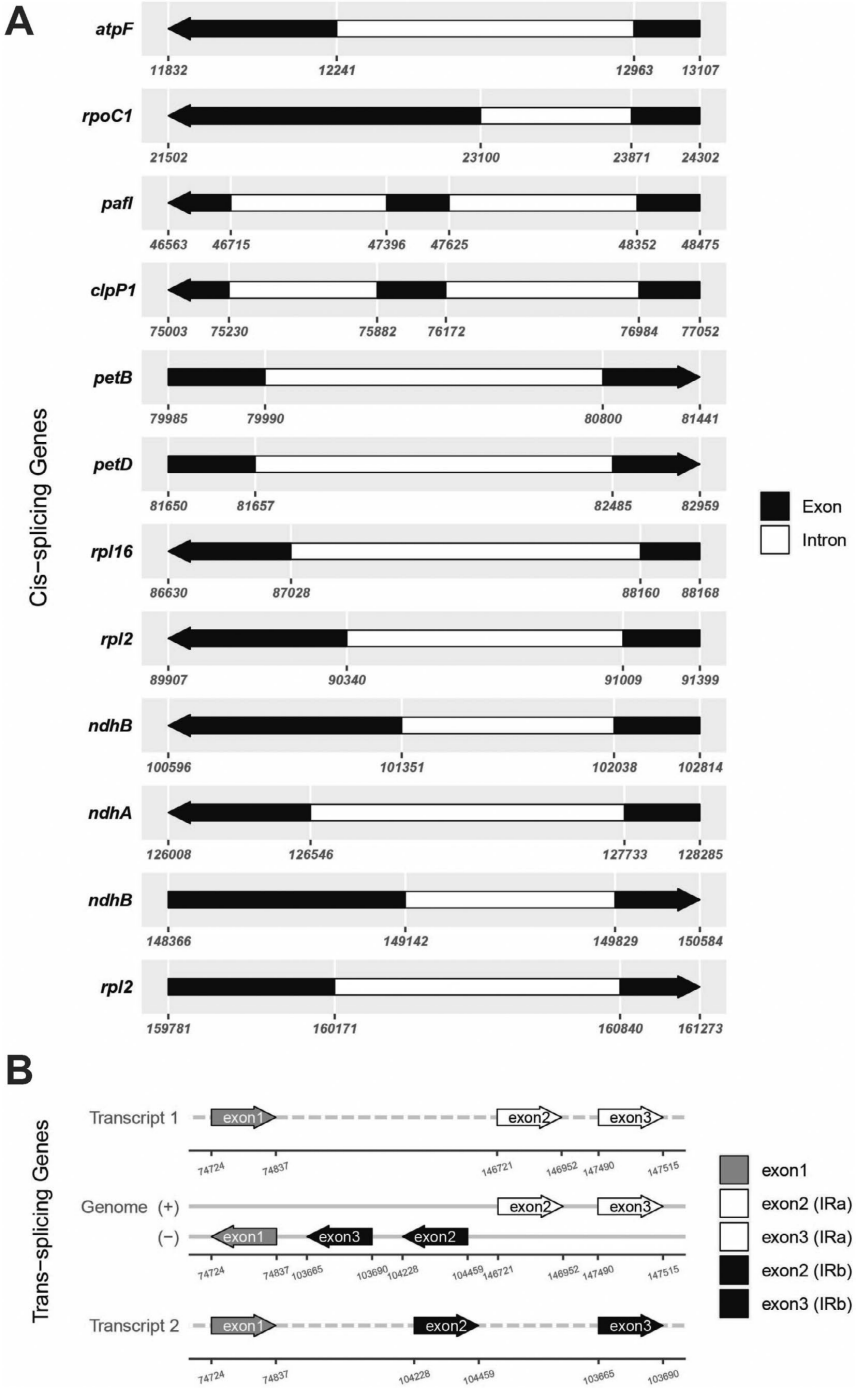
Schematic map of the cis and trans‐splicing genes in the chloroplast genomes of *Baccaurea ramiflora* cultivar Ha Chau. (A) The cis‐splicing genes. (B) The trans‐splicing genes.

A total of 589,122 reads out of 32,296,794 reads were assembled to complete the chloroplast genome of *B. ramiflora* cultivar Ha Chau with a coverage depth of 546×. The size of the chloroplast genome was 161,378 bp, including a large single copy (LSC, 89,801 bp), a small single copy (SSC, 18,833 bp), and two inverted repeat (IR, 26,372 bp each) regions (Table 2). This circular genome contained 79 protein‐coding genes, 30 tRNA genes, and four rRNA genes (Figure 2, Table 2). There were 20 duplicated genes in the IR regions, including *rrn4.5*, *rrn5*, *rrn16*, *rrn23*, *trnA‐UGC*, *trnL‐CAA*, *trnI‐GAU*, *trnI‐CAU*, *trnN‐GUU*, *trnR‐ACG*, *trnV‐GAC*, *rpl2*, *rpl22*, *rpl23*, *rps7*, *rps12*, *rps19*, *ndhB*, *ycf1*, and *ycf2*, of which *ycf1* and *rps19* were incompletely duplicated. Among annotated regions, *trnA‐UGC*, *trnG‐UCC*, *trnI‐GAU*, *trnK‐UUU*, *trnL‐UAA*, *trnV‐UAC*, *rpl2*, *rpl16*, *rps16*, *rpoC1*, *petB*, *petD*, *atpF*, *ndhA*, and *ndhB* had one intron, whereas *pafI* and *clpP1* had two introns (Figure 3A). The *rps12* gene was a trans‐splicing gene, of which exon 1 was in the LSC region, meanwhile exon 2 and exon 3 were located in the IR regions (Figure 3B). Notably, exon 1 of *rps16* was absent and the *infA* gene contained internal stop codons, resulting in the pseudogenization of these two genes in the chloroplast genome of *B. ramiflora* cultivar Ha Chau.

**TABLE 2:** Gene composition of *Baccaurea ramiflora* cultivar Ha Chau chloroplast genome.

Groups of genes	Name of genes
Ribosomal RNAs	*rrn4.5* ^a^, *rrn5* ^a^, *rrn16* ^a^, *rrn23* ^a^
Transfer RNAs	*trnA‐UGC* ^a^, ^b^, *trnC‐GCA, trnD‐GUC, trnE‐UUC, trnF‐GAA, trnG‐UCC* ^b^, *trnG‐GCC, trnH‐GUG, trnI‐GAU* ^a^, ^b^, *trnK‐UUU* ^b^, *trnL‐CAA* ^a^, *trnL‐UAA* ^b^, *trnL‐UAG, trnfM‐CAU, trnI‐CAU* ^a^, *trnM‐CAU, trnN‐GUU* ^a^, *trnP‐UGG, trnQ‐UUG, trnR‐ACG* ^a^, *trnR‐UCU, trnS‐GCU, trnS‐GGA, trnS‐UGA, trnT‐GGU, trnT‐UGU, trnV‐GAC* ^a^, *trnV‐UAC* ^b^, *trnW‐CCA, trnY‐GUA*
Large units of ribosome	*rpl2* ^a^, ^b^, *rpl14, rpl16* ^b^, *rpl20, rpl22* ^a^, *rpl23, rpl32, rpl33, rpl36*
Small units of ribosome	*rps2, rps3, rps4, rps7* ^a^, *rps8, rps11, rps12* ^a^, ^c^, *rps14, rps15, rps16* Ψ, *rps18, rps19* ^a^
RNA polymerase	*rpoA, rpoB, rpoC1* ^b^, *rpoC2*
Translational initiation factor	*infA*Ψ
Subunit of photosystem I	*psaA, psaB, psaC, psaI, psaJ, pafI* ^c^, *pafII*
Subunit of photosystem II	*psbA, psbB, psbC, psbD, psbE, psbF, psbH, psbI, psbJ, psbK, psbL, pbfI, psbM, psbT, psbZ*
Subunit of cytochrome	*petA, petB* ^b^, *petD* ^b^, *petG, petL, petN*
Subunit of ATP synthases	*atpA, atpB, atpE, atpF* ^b^, *atpH, atpI*
Large unit of Rubisco	*rbcL*
Subunit of NADH dehydrogenase	*ndhA* ^b^, *ndhB* ^a^, ^b^, *ndhC, ndhD, ndhE, ndhF, ndhG, ndhH, ndhI, ndhJ, ndhK*
Maturase	*matK*
Envelope membrane protein	*cemA*
Subunit of acetyl‐CoA	*accD*
C‐type cytochrome synthesis gene	*ccsA*
ATP‐dependent protease subunit P	*clpP1* ^c^
Hypothetical proteins and conserved reading frames	*ycf1* ^a^, *ycf2* ^a^

^a^ Duplicated gene in IR region.

^b^ Genes containing single intron.

^c^ Genes containing two introns, Ψ pseudogene.

Comparative analysis of chloroplast genomes of different *B. ramiflora* individuals revealed a conservation trend regarding genome structure and gene content (Table 3). Although all examined species had 79 protein‐coding genes, 30 tRNA genes, and four rRNA genes, their sizes were slightly different, which ranged from 160,770 to 161,378 bp. Consequently, the lengths of LSC, SSC, and IR regions fluctuated from 89,452 to 89,901 bp, from 18,818 to 18,833 bp, and from 26,246 to 26,400 bp, respectively. The junction between SSC and IR regions was within *ycf1* in all surveyed *B. ramiflora* individuals. However, the boundaries between LSC and IR regions varied slightly. Although the junction was located in the *rps19* gene, the junction sites shifted from 43 to 78 bp from the start codon of the gene. One exception was the location within the intergenic space between *rpl2* and *trnH‐GUG* for LSC/IRA junction and within the intergenic space between *rps19* and *rpl2* for LSC/IRB junction in the record of ON881834 (Table 3). Further pairwise identity analysis demonstrated a high genomic diversity of *B. ramiflora* cultivar Ha Chau in comparison with other *B. ramiflora* individuals. Specifically, the pairwise identities between cultivar Ha Chau and other *B. ramiflora* chloroplast genomes were lower than 98.8%. Meanwhile, the similarities of other *B. ramiflora* chloroplast genomes were greater than 99%. These differences might indicate a unique genomic evolution of *B. ramiflora* cultivar Ha Chau that provides new insights into the evolutionary history of the *Baccaurea* genus and needs further genomic studies.

**TABLE 3:** Comparative genomic features of *Baccaurea ramiflora* cultivar Ha Chau and related species.

Species	*B. ramiflora*	*B. ramiflora*	*B. ramiflora*	*B. ramiflora*	*B. ramiflora*
Accession number	PQ469841	PP497828	MT900598	MW717296	ON881834
Total length (bp)	161,378	161,183	161,093	161,089	160,770
Total %GC	36.7	36.7	36.7	36.7	36.7
LSC length (bp)	89,801	89,560	89,503	89,515	89,452
LSC % GC	34.3	34.4	34.4	34.4	34.4
SSC length (bp)	18,833	18,823	18,818	18,826	18,826
SSC % GC	30.8	30.8	30.8	30.8	30.8
IR length (bp)	26,372	26,400	26,386	26,374	26,246
IR % GC	42.7	42.7	42.7	42.7	42.8
Protein‐coding gene	79	79	79	79	79
tRNAs	30	30	30	30	30
rRNAs	4	4	4	4	4
LSC/IRA junction	*rps19* (43 bp)	*rps19* (77 bp)	*rps19* (78 bp)	*rps19* (78 bp)	IGS (*rpl2/trnH_GUG*)
LSC/IRB junction	*rps19* (43 bp)	*rps19* (77 bp)	*rps19* (78 bp)	*rps19* (78 bp)	IGS (*rps19/rpl2*)
SSC/IR junction	*ycf1* (955 bp)	*ycf1* (955 bp)	*ycf1* (955 bp)	*ycf1* (955 bp)	*ycf1* (955 bp)
*Pairwise identity (%)*					
Species	**PQ469841**	PP497828	MT900598	MW717296	ON881834
**PQ469841**	100	98.757	98.788	98.771	98.766
PP497828	98.753	100	99.107	99.056	99.181
MT900598	98.788	99.107	100	99.880	99.884
MW717296	98.771	99.056	99.880	100	99.811
ON881834	98.766	99.181	99.884	99.811	100

*Note:* Bold words indicate *B. ramiflora* cultivar Ha Chau.

In general, the complete chloroplast genome of *B. ramiflora* cultivar Ha Chau was similar to those of Phyllanthaceae members such as *B. ramiflora* , *Leptopus cordifolius*, *Phyllanthus acidus* , and *Flueggea virosa* regarding genome structure and gene content (Hu et al. 2021; Rehman et al. 2021; Niu and Liu 2022; Nguyen et al. 2023). However, a notable genomic event in the chloroplast genome of *B. ramiflora* was the absence of *rps16* exon1. Further investigation of *rps16* status in Phyllanthaceae indicated that there were four stages, including intact, completely lost, exon1 remained, and exon2 remained (Table 1). Furthermore, the subfamily Antidesmatoideae had intact and exon2 remained *rps16* whereas the subfamily Phyllanthoideae had intact, completely lost, and exon2 remained *rps16*. Previous reports indicate the loss of *rps16* in different plants of Euphorbiaceae and Fabaceae (Keller et al. 2017; Zhang et al. 2021; Li et al. 2022). The continuous deletion of *rps16* was also recorded in Melanthiaceae members (Do and Kim 2017). These results revealed a dynamic evolution of *rps16* in land plants. In this study, 14 out of 59 genera of Phyllanthaceae were used to explore the variation of *rps16*. Therefore, further research covering more samples of Phyllanthaceae should be conducted to find the variation patterns of *rps16*, which might be useful for developing molecular markers for identification.

**FIGURE 4:**
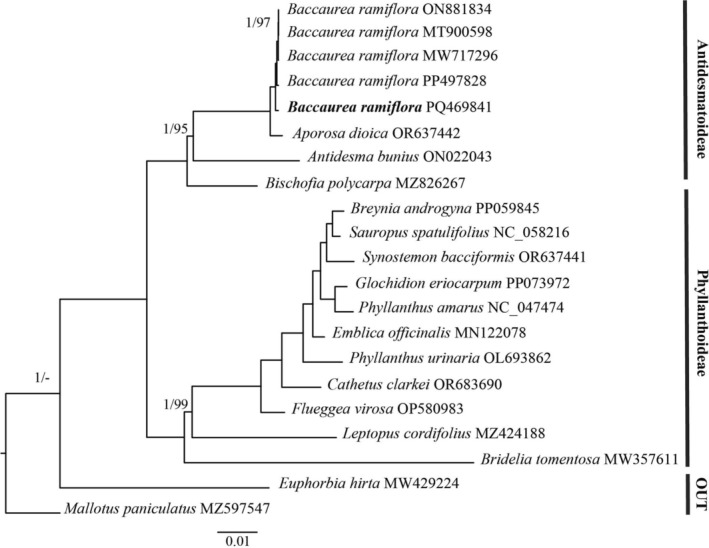
Phylogenetic relationship of *Baccaurea ramiflora* cultivar Ha Chau and related species inferred from 77 protein‐coding genes using maximum likelihood and Bayesian Inference methods. Only posterior probability under 1 and bootstrap values smaller than 100 were shown at the nodes. The dash means no value.

For phylogenetic analysis, the ML and BI methods inferred from 77 protein‐coding genes resulted in the same topological phylogenetic trees (Figure 4). The samples of *B. ramiflora* formed a clade that is sister to *Aporosa dioica* with high support values (bootstrap value = 100 and posterior probability = 1). Additionally, the monophyly of the two subfamilies Antidesmatoideae and Phyllanthoideae was confirmed. These findings are in agreement with previous phylogenetic studies based on gross morphology and molecular data (Kathriarachchi et al. 2005; Moustafa et al. 2024).

## 4 Conclusion

In this study, we sequenced and characterized the complete chloroplast genome of *B. ramiflora* cultivar Ha Chau using the next‐generation sequencing method. The newly completed chloroplast genome is similar to those of other *B. ramiflora* individuals in terms of genome structure and gene content. Phylogenetic analysis inferred from 77 protein‐coding regions indicated a close relationship among *B. ramiflora* individuals as well as the monophyly of two subfamilies of Phyllanthaceae. Comparative genomic analyses revealed a high variation of the *B. ramiflora* cultivar Ha Chau chloroplast genome in comparison to its counterparts, which provided essential genomic data for further studies examining the evolutionary history of the *Baccaurea* genus and related species in Phyllanthaceae.

## Author Contributions

**Pham Anh Thi Nguyen:** conceptualization (equal), data curation (lead), formal analysis (equal), writing – original draft (lead). **Hanh An Nguyen:** data curation (supporting), methodology (supporting), software (supporting), visualization (equal), writing – review and editing (supporting). **Hoang Dang Khoa Do:** conceptualization (equal), data curation (supporting), investigation (lead), writing – review and editing (supporting). **Do Tan Khang:** conceptualization (equal), formal analysis (equal), methodology (lead), software (lead), writing – review and editing (equal).

## Conflicts of Interest

The authors declare no conflicts of interest.

## Data Availability

The complete chloroplast genome of *Baccaurea ramiflora* was submitted to GenBank under accession number PQ469841. The raw sequencing data were also deposited to NCBI under the accession number PRJNA1203916.
